# Rapid Detection of Alpha-Fetoprotein (AFP) with Lateral Flow Aptasensor

**DOI:** 10.3390/molecules30030484

**Published:** 2025-01-22

**Authors:** Meijing Ma, Min Zhang, Jiahui Wang, Yurui Zhou, Xueji Zhang, Guodong Liu

**Affiliations:** 1 School of Chemistry and Chemical Engineering, Linyi University, Linyi 276005, China; 220703001552@lyu.edu.cn (M.M.); 220703001572@lyu.edu.cn (M.Z.); wangjiahui1@lyu.edu.cn (J.W.); zhouyurui@lyu.edu.cn (Y.Z.); 2 Marshall Laboratory of Biomedical Engineering, Research Center for Biosensor and Nanotheranostic, School of Biomedical Engineering, Shenzhen University, Shenzhen 518060, China

**Keywords:** aptamer, AFP, gold nanoparticles, lateral flow, biosensors, visual detection

## Abstract

We present a lateral flow aptasensor for the visual detection of alpha-fetoprotein (AFP) in human serum. Leveraging the precise molecular recognition capabilities of aptamers and the distinct optical features of gold nanoparticles, a model system utilizing AFP as the target analyte, along with a pair of aptamer probes, is implemented to establish proof-of-concept on standard lateral flow test strips. It is the first report of an antibody-free lateral flow assay using aptamers as recognition probes for the detection of AFP. The analysis circumvents the numerous incubation and washing steps that are typically involved in most current aptamer-based protein assays. Qualitative analysis involves observing color changes in the test area, while quantitative data are obtained by measuring the optical response in the test zone using a portable strip reader. The biosensor exhibits a linear detection range for AFP concentrations between 10 and 100 ng/mL, with a minimum detection limit of 10 ng/mL. Additionally, it has been successfully applied to detect AFP in human serum samples. The use of aptamer-functionalized gold nanoparticle probes in a lateral flow assay offers great promise for point-of-care applications and fast, on-site detection.

## 1. Introduction

Hepatocellular carcinoma (HCC), a common form of cancer globally, is frequently the main cause of death in patients diagnosed with cirrhosis [1]. As is well known, early diagnosis and early-stage treatment is the main method to reduce cancer mortality. The majority of contemporary clinical techniques, including imaging and histology, are only applicable in the context of advanced HCC. The use of serum tumor biomarker testing techniques can facilitate more accurate diagnosis of associated tumors [2]. In recent years, alpha-fetoprotein (AFP) has garnered significant attention and has emerged as the most widely utilized protein biomarker for diagnosing hepatocellular carcinoma (HCC) [3,4]. AFP is primarily produced by the yolk sac during fetal development and later secreted by the liver at around 7–8 months of gestation [5,6]. Its levels gradually decrease as fetal development progresses. In healthy individuals, serum AFP concentrations typically remain below 25 ng/mL [7]. However, approximately 75% of patients with hepatocellular carcinoma (HCC) exhibit significantly elevated AFP levels, often reaching as high as 500 ng/mL. In adults, increased AFP levels in the bloodstream can serve as a biomarker for various cancers, including those of the liver, stomach, pancreas, ovaries, and testicles [8,9].

In recent years, the detection of AFP based on lateral flow biosensors has gained significant attention and application [10,11]. A number of AFP test kits based on the lateral flow assay have emerged domestically and internationally, with the detection limits typically within the range of 10 to 25 ng/mL (see details in Table S1 of Supplementary Materials). To improve the sensitivity of the detection, researchers have been developing new nanomaterials to replace the traditional colloidal gold. Furthermore, fluorescence [12], Raman [13], chemiluminescence [14], near-infrared light [15], and other methods have been used as detectors to obtain a more sensitive signal than the traditional colorimetric method. Given the distinctive attributes of cancer biomarkers, the receptor, serving as the recognition element for the target molecule, is pivotal for the precise and sensitive detection of cancer biomarkers. Nevertheless, the aforementioned AFP lateral flow test strips are all based on the double-antibody sandwich-type method of antibody–antigen immunoreactions. However, the instability of the antibody and the complexity of the manipulation result in high costs and low efficiency. In this context, aptamers that can recognize a broad spectrum of targets—including oligonucleotides, proteins, small molecules, exosomes, and even entire cells—have been developed and are extensively used in biosensor design for the early detection of various cancer biomarkers [16,17,18].

Nucleic acid aptamers are a class of oligonucleotide molecules that exhibit high affinity and strict specificity for target molecules [19]. These were distinguished using the Exponential Enrichment Ligand System Evolution (SELEX) method, which involves screening artificially constructed oligonucleotide libraries [20]. The aptamer is maneuverable, reproducible, easy to immobilize and regenerate, and shows consistency across different batches. It has seen widespread applications in sensor technology. Huang et al. reported a SELEX/microfluidic chip method to screen an AFP-specific aptamer [21]. Dong et al. discovered an AFP-specific single-stranded DNA aptamer, AP273, through the SELEX process combined with capillary electrophoresis [22]. A variety of aptamer-based biosensors have been designed for AFP detection, employing methods like Raman spectroscopy, dual-polarization interferometry, resonance light scattering, fluorescence, chemiluminescence, cyclic voltammetry, electrochemical impedance, and the giant magneto-resistive technique [23]. However, those reported aptamer-based biosensors for the detecting of AFP require multiple incubation and washing steps, expensive instruments, and skilled personnel, which limit its applications in point-of-care testing [23]. In this paper, we report a lateral flow aptasensor for the qualitative (visual) detection of AFP in 10 min. It is the first report of an antibody-free lateral flow assay using aptamers as recognition probes for the detection of AFP. The biosensor was successfully applied for the detection of AFP in human whole serum samples with satisfactory results.

## 2. Results

### 2.1. Characterization of AuNP and AuNP-Det-Apt Conjugates

The synthesized AuNPs were characterized using transmission electron microscopy (TEM), nanoparticle size and zeta potential analysis, and ultraviolet–visible spectroscopy (UV-vis), respectively (Figure S1 in Supplementary Materials). Figure S1A depicts a representative transmission electron microscopy (TEM) image of gold nanoparticles (AuNPs). Figure S1B shows the particle size distribution of the AuNPs. Figure S1A,B demonstrate the successful preparation of 25 nm gold nanoparticles. Figure S1C displays the ultraviolet (UV) spectra of the gold nanoparticles before and after conjugation with the AFP aptamer. The appearance of the characteristic peak of DNA at 263 nm indicates the successful conjugation of the AFP aptamer with AuNP [24]. Gel electrophoresis was also used to confirm that the aptamer was conjugated with the AuNP (Figure S2 in Supplementary Materials). The details of these characterizations can be found in Supplementary Materials.

### 2.2. Working Principles of the AuNP-Based Lateral Flow Aptasensor

**Figure 1:**
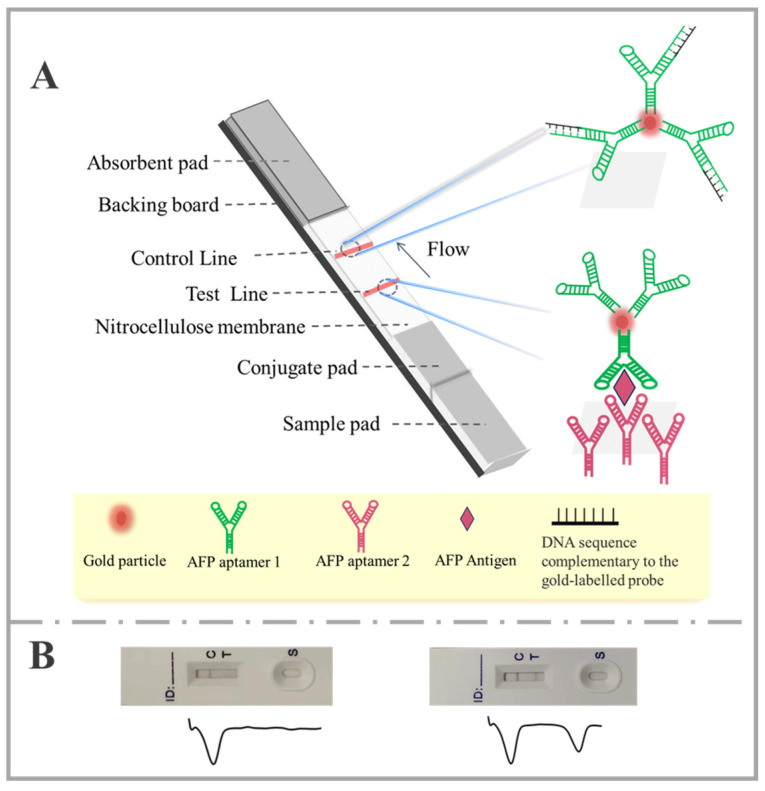
(**A**) Schematic illustration of the structure and measurement principle of the lateral flow aptasensor. (**B**) Pictures (top) and corresponding responses from colloidal gold analyzer (bottom) of the tested strips in the absence (left) and presence of AFP (right).

Figure 1 provides a schematic overview of the structure and working principle of lateral flow aptasensor, which comprises five components, a sample pad, a conjugate pad, a nitrocellulose membrane, and an absorbent pad, that are all mounted on a common substrate layer (typically a polyvinyl chloride material) (Figure 1A). Typically, an AFP sample solution is added to the sample pad. Afterward, the solution is transported by capillary action, enabling the rehydration of aptamer (AFP aptamer 1) on the AuNP-Det-Apt conjugates (AuNP-Det-Apt refers to the complex formed by AuNPs and the aptamer detection probe. “Det” is the abbreviation of “detection”. (The detection probe refers to the aptamer probe that is conjugated with AuNP.) Subsequently, the AuNP-Det-Apt binds to the AFP, forming a complex that migrates along the strip. When this complex reaches the test zone, it is captured by the Cap-Apt (AFP aptamer 2), which is immobilized on the test zone through the interaction of the biotinylated aptamer and streptavidin. The accumulation of AuNPs in the test area results in the formation of a distinctive red band. The phenomenon of capillary action then causes the liquid sample to migrate further. An excess of the AuNP-Det-Apt conjugate is retained in the control region through hybridization between the quality control DNA probe, pre-immobilized in this region, and Det-Apt. This interaction produces the second red band. When AFP is absent, a red band appears only in the control region, with no band forming in the test region. In this case, the presence of a red band in the control zone (control line) verifies the proper operation of the biosensor. Qualitative analysis was carried out by monitoring color changes specifically in the test region, while quantitative analysis involved measuring the red band’s light intensity using a portable colloidal gold analyzer. The peak intensity’s height is directly linked to the quantity of AuNPs captured in the test region and correlates with the AFP concentration in the sample. Figure 1B presents the typical photo images (top) and corresponding responses from colloidal gold analyzer (bottom) of the tested strips in the absence (left) and presence of AFP (right).

### 2.3. Optimization of Analytical Parameters

The preparation and experimental parameters of aptamer-based lateral flow biosensors have a significant impact on its analytical performance. To achieve optimal reproducibility and sensitivity, we optimized several parameters: (a) the AuNP concentration, (b) the type of nitrocellulose membrane, (c) the concentration of aptamers during conjugation, (d) the volume of AuNP-Det-Apt conjugates on the conjugate pad, (e) the volume of the running buffer, and (f) the volume of streptavidin-biotinylated Cap-Apt (capture probe aptamer) in the test zone. The analytical performance of lateral flow aptasensor was assessed using the signal-to-noise ratio (S/N), with one condition optimized at a time while keeping the others constant. A comprehensive analysis of the collected data, along with the relevant graphs, is provided in Figure S3. The experimental conditions used to achieve the best results were as follows: (a) the optimal concentration of AuNP is 16 OD, (b) VIVID nitrocellulose membranes were found to be the most effective, (c) the optimal concentration of the aptamer during conjugation is 8 μmol, (d) the optimal volume of conjugate required at each strip conjugate pad is 1.3 µL, (e) a volume of 50 µL of running buffer has been found to be optimal, (f) the volume of streptavidin-biotinylated cap-DNA present within the designated test area is 1.5 µL. A detailed discussion of the optimizations is provided in the Supplementary Materials.

### 2.4. Analytical Performance

**Figure 2:**
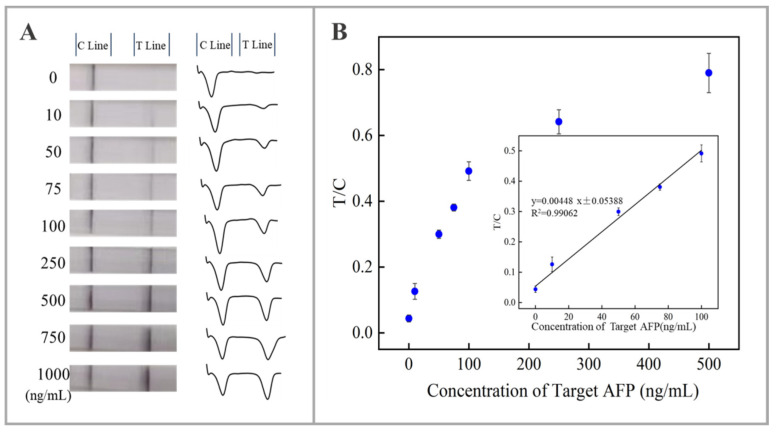
(**A**) Displays photographs and corresponding profile plots of lateral flow aptasensors at different AFP concentrations. (**B**) Dot plots and calibration curve (inset) for the detection of AFP under optimal experimental conditions.

The analytical performance of the aptamer-based lateral flow biosensor was evaluated by testing different AFP concentrations under the optimal experimental parameters. Figure 2A presents the photo images and the corresponding optical responses of the tested strips for the detection of different concentrations of AFP. One can see the captured AuNPs aggregated in the test area, forming visible bands for qualitative analysis. The density of the test bands increases with the increase in AFP concentration. The control group, which served as the blank experimental group, had an AFP concentration of 0 ng/mL. The test region of the control group exhibited no discernible color band, indicating that non-specific adsorption was not a significant phenomenon under the optimized experimental conditions. It is noted that even at an AFP concentration of only 10 ng/mL, a weak detection band could still be discerned.

For quantitative analysis, a portable colloidal gold analyzer was used to determine the band density, which correlated with the concentration of AFP. Figure 2B illustrates that as the concentration of the AFP increases, the signal-to-noise ratio (T/C) also increases gradually. The inset illustrates the calibration curve of the lateral flow aptasensor for the detection of AFP, demonstrating that the biosensor exhibits a satisfactory linear dynamic range between 0 and 100 ng/mL. The calibration equation, y = (0.00448)x + (0.05388 ± 0.01357), was derived by linear fitting, where y represents the T/C ratio and x represents the AFP concentration. The equation has a correlation coefficient R^2^ of 0.99062 and a calculated detection limit of 7.08 ng/mL (S/N = 3).

### 2.5. Specificity Tests

**Figure 3:**
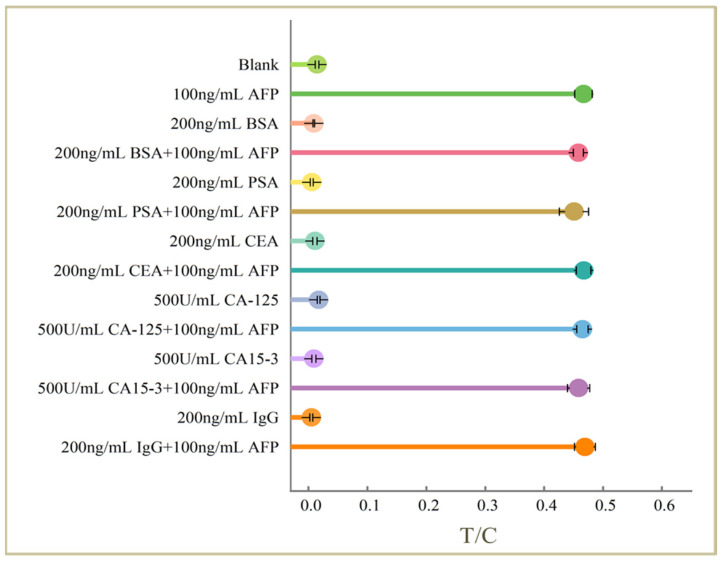
Specificity test results of the lateral flow aptasensor.

In order to evaluate the specificity of the lateral flow aptasensor for AFP detection, a series of high concentrations of proteins including bovine serum albumin (BSA), prostate cancer antigen (PSA), carcinoembryonic antigen (CEA), ovarian cancer-associated antigen (CA-125), breast cancer-associated antigen (CA15-3), and immunoglobulin G (IgG) were selected for validation. Figure 3 presents the results of specificity tests. The pictures taken during the experiment and the corresponding optical responses can be found in Figure S4 of Supplementary Materials. One can see that negligible response was obtained in the presence of the selected proteins at high concentration. In addition, we measured the responses of the mixture of AFP and the selected proteins on the lateral flow aptasensor. AFP with a final concentration of 100 ng/mL was prepared by spiking AFP to each of the selected protein. It can be seen that there is no significant difference of the response in the presence and absence of the selected proteins, indicating the lateral flow aptasensor had good specificity.

### 2.6. Testing of Actual Samples

**Figure 4:**
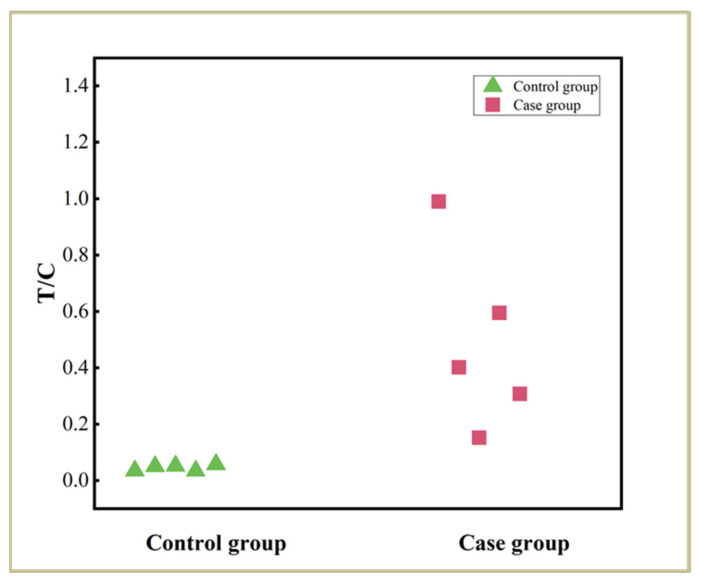
Test results of 10 serum samples.

In order to assess the potential of the lateral flow aptasensor for clinical applications, five healthy human serum samples and five liver cancer patient serum samples were tested with the lateral flow aptasensors. All serum samples were provided by Linyi First People’s Hospital with informed consent. Seventy microliters of whole serum samples were transferred to the sample wells of the lateral flow aptasensors, and the test results were obtained after waiting 15 min. The pictures taken during the experiment and the corresponding optical responses can be found in Figure S5 of the Supporting Information. As illustrated in Figure 4, the responses of the biosensor from healthy individuals are significantly lower than those from liver cancer patients. These findings highlight the significant potential of the developed biosensor used for screening liver cancer.

## 3. Discussion

This article presents the successful development of a lateral flow aptasensor for the detection of AFP with a limit of detection of 10 ng/mL by the naked eye. Furthermore, the sensor has been successfully applied to the detection of human whole serum, which can be attributed to the selection of a pair of aptamers with good affinity. The most commonly reported and applied AFP aptamers in biosensing assays are AP-Taiwan [21], AP-273 [22,25,26,27,28], and AP-3 [29,30], with the specific sequences presented in Table 1. In 2011, Huang et al. identified a specific aptamer (AP-Taiwan) with a Kd value of 2.37 nM [21]. This is comparable to the affinity of the AFP antibody (10^−7^~10^−9^ M) [31]. In 2015, Dong et al. identified the aptamer (AP-273) and found that the sequence could be folded into two conformations [22]. Subsequently, they calculated the Kd values of AP-273 and AP-Taiwan with AFP. The Kd values of AP-273 and AP-Taiwan with AFP proteins were 17.41 ± 4.32 nM and 18.89 ± 5.44 nM, respectively. The sequence of AP-3 is identical to that of the middle portion of AP-273 (see the bold section in Table 1), and thus it is frequently employed for the detection of AFP proteins [29,30]. Accordingly, in the course of our experiments, we employed a variety of combinations of the aptamers derived from these three sequences and subjected them to compare their analytical performances. Each of the three aptamers was conjugated with AuNP. Meanwhile, each of the three aptamers was used as the test (T) line, forming nine different combinations. When AP-273 was used both as the conjugate probe and the capture probe at the T line, AFP at a concentration of 100 ng/mL could be detected. For the other several combinations, there were no obvious phenomena. Ultimately, we selected AP-273 as both the detection and capture probes for subsequent exploration and optimization, likely due to its superior affinity and the fact that it exhibits two conformations.

Dong et al. identified the aptamer (AP-273) and found that the sequence of AP-273 could be folded into two conformations [22]. An aptamer with a different conformation may specifically recognize and bind to spatially separated different sites on AFP. This enables two aptamers to bind to AFP simultaneously, with each aptamer interacting with its respective target site on the protein, thus forming a “sandwich-type” structure with AFP in the middle. During conjugation, the phenomenon of induced fit may occur. The initial interaction between aptamer and AFP leads to a conformational change in the aptamer that enhances the binding affinity and specificity. This conformational change can also enable the second aptamer to bind more effectively to its corresponding target site on AFP, facilitating the formation of a stable “sandwich” complex. In addition, the appearance of the characteristic peak at 263 nm in the ultraviolet absorption spectrum of the conjugate of AuNP and aptamer shown in Figure S1C, as well as the pictures of the changes before and after the conjugation of AuNP and aptamer in the agarose gel electrophoresis test in Figure S2, can confirm the successful preparation of the AuNP-Apt conjugates. Subsequently, the conjugate was sprayed onto the conjugate pad. Meanwhile, another aptamer was immobilized at the test line (T line) of the test strip. When AFP is introduced, AFP will first bind to the conjugate. Then, the same aptamer at the T line will bind to the formed AuNP-Apt-AFP, forming a “sandwich-type” complex: AuNP-Apt-AFP-Apt. Therefore, the color signal of the colloidal gold at the T line can confirm the formation of the “sandwich-type” structure.

In the serum of healthy individuals, the concentration of AFP is less than 25 ng/mL [7]. Notably, in nearly 75% of HCC patients, the AFP concentration rises remarkably to 500 ng/mL [8]. Currently, the lateral flow biosensors reported for AFP detection all operate on the principle of two antibodies capturing AFP (Table S2 in Supplementary Materials). In the pursuit of enhanced sensitivity and specificity, researchers have been developing antibody-based lateral flow AFP biosensors based on novel materials or signal readers in recent years. However, the aptamer-based lateral flow biosensor for AFP detection has yet to be documented. The antibody-based lateral flow assay for AFP detection has been widely commercialized, with a detection limit typically ranging from 10 ng/mL to 25 ng/mL (Table S1 in Supplementary Materials). The aptamer-based lateral flow biosensor developed in this study has a detection limit of 10 ng/mL, which can fulfill the detection requirements.

The experiment revealed that combining the up-sampling buffer with healthy human serum and introducing it into the spiking wells resulted in a false-positive background signal. As the ionic strength of the up-sampling buffer was increased, the background signal was subsequently reduced, and the sensitivity was also decreased. However, when only healthy human serum is added without buffer, no false-positive signal is observed, indicating that the addition of serum has an interfering effect on the buffer system. Consequently, during real sample testing, the decision was taken to test whole serum without the addition of buffer. Further investigation is required to elucidate the impact of serum on the buffer, with a view to addressing this issue in future research.

Additionally, we observed that the test strip exhibited purple-black bands rather than the traditional red bands associated with 25 nm AuNPs. This phenomenon may be attributed to the aggregation of AuNPs following the resuspension of the AuNP-Apt conjugate in the resuspension buffer. Initially, we observed a color change when the AuNP-Apt conjugate was suspended in various aqueous solutions. As depicted in Figure S6A, the AuNP-Apt conjugate appeared red when suspended in water but turned purple-black upon suspension in the resuspension buffer. The alteration in the conjugate solution indicates the aggregation of AuNP-Apt. We measured the particle size of the AuNP-Apt conjugate using a nanoparticle size and zeta potential analyzer. Figure S6B illustrates that the average particle size of AuNPs increased to 99 nm after the AuNP-Apt conjugate was resuspended in the buffer. The aggregation of the AuNP-Apt conjugate in the resuspension buffer was further confirmed using transmission electron microscopy (TEM). As shown in Figure S6C, the AuNPs had agglomerated. Concurrently, we optimized the loading buffer during the lateral flow assay. As depicted in Figure S7, black bands appeared in various buffer systems, including HEPES, PBS, Tris-HCl, and SSC. Moreover, the 250 ng/mL AFP was only detected in the HEPES buffer system. The aforementioned results indicate that the black bands on the test strip result from agglomeration and increased particle size of the AuNPs. This also suggests that the conjugation between AFP and the aptamer necessitates a specific environment.

## 4. Materials and Methods

### 4.1. Apparatus

An X-Y-Z three-dimensional film spraying instrument (HM3035), microcomputer automatic chopper (ZQ2002), and CNC strip cutting machine (CTS300) were purchased from Shanghai Gold Standard Biotechnology Co., Ltd. (Shanghai, China); freezing centrifuge (DH-20KR) was purchased from GallopTech Co., Ltd. (Shanghai, China); nanoparticle size and zeta potential analyzer (BeNano 90 Zeta) was bought from Dandong Better size Instruments Ltd. (Dandong, China); gel imaging system was purchased from (GS-500) GallopTech Instruments Shanghai Co., Ltd. (Shanghai, China); ultrapure water analyzer (Simple Q30) was bought from ZhiAng Instruments Shanghai Co., Ltd. (Shanghai, China); high-temperature oven (DHG9023A) was purchased from Shanghai Qixin Scientific Instruments Co., Ltd. (Shanghai, China); ultraviolet spectrophotometer (UV-6100) was bought from Shanghai Metash Instruments Co., Ltd. (Shanghai, China); and colloidal gold analyzer (GIC-S100) was purchased from Suzhou Hexiang Instruments Co., Ltd. The transmission electron microscope used in the article was purchased from Hitachi (HT7800).

### 4.2. Reagents and Materials

All reagents used in this study were of analytical grade, and all solutions were prepared with ultrapure water possessing a resistivity of 18.25 MΩ·cm. Gold(III) chloride trihydrate (HAuCl_4_·3H_2_O, 99.9%) was obtained from Sigma-Aldrich, Inc. (St. Louis, MO, USA). Triton X-100, magnesium chloride (MgCl_2_), Tween-20, trisodium citrate (Na_3_Ct), n-butanol, sodium phosphate dodecahydrate (Na_3_PO_4_·12H_2_O), and 6-mercapto-1-hexanol (MCH) were purchased from Shanghai Macklin Biochemical Science and Technology Co., Shanghai, China. Sodium chloride, dextrose, and sucrose were purchased from Sinopharm Chemical Reagent Co., Beijing, China. Bovine serum albumin (BSA), Tris-hydrochloric acid (Tris-HCl), and 4-hydroxyethylpiperazine ethane sulfonic acid (HEPES) were purchased from Beijing Solarbio Technology Co., Beijing, China. Absorbent paper was purchased from Shanghai Goldbio technology Co. Ltd., Shanghai, China, and glass fiber and nitrocellulose membranes were purchased from Shanghai Jinning Biotechnology Co., (Shanghai, China).

All oligonucleotides used in this study were purchased from Sangon Biotech (Shanghai, China). The nucleotide sequence of the aptamer detection probe (Det-Apt) was as follows: SH-GTGACGCTCCTAACGCTGACTCAGGTGCAGTTCTCGACTCGGTCTTGATGTGGGTCCTGTCCGTCCGAACCAATC.

The sequence of the capture probe (Cap-Apt) was as follows: Biotin-GTGACGCTCCTAACGCTGACTCAGGTGCAGTTCTCGACTCGGTCTTGATGTGGGTCCTGTCCGTCCGAACCAATC.

The quality control probe DNA sequence was Biotin-GATTGGTTCGGACG.

### 4.3. Preparation of Gold Nanoparticles (AuNPs)

AuNPs were prepared at a concentration of 4 parts per million, in accordance with the methodology previously outlined in the literature with minor modification [32]. All glassware used in the experiments was soaked in aqua regia overnight before use and rinsed with double-distilled water until clean. In a conical flask, 192 mL of ultrapure water (18.25 MΩ·cm) was added into 8 mL of a 1% gold (III) chloride trihydrate solution. The solution was heated and stirred until boiling point was reached. A single rapid addition of 4.7 mL of a 2% trisodium citrate solution was made, resulting in a change from a light yellow to a blue-black and finally burgundy coloration. After the solution was boiled for additional ten minutes, the heat source was removed. When the temperature reached room temperature, the colloidal gold solution obtained was kept at 4 °C.

### 4.4. Preparation of AuNP-Det-Apt Conjugates

Before conjugation, the colloidal gold solution was concentrated to one-eighth of its original volume using a centrifuge at a speed of 8000 revolutions per minute. Specifically, 400 μL of the colloidal gold solution was taken, and after centrifugation to remove the supernatant, 50 μL of water was added for resuspension. The concentrated was then conjugated with a sulfhydryl-modified Det-Apt using a butanol dehydration method [33], using the following procedure: The concentrated colloidal gold solution (50 μL) was mixed with the Det-Apt (4 μL, 100 μM) and n-butanol solution (900 μL). The mixture was then shaken for 20–30 s using a palmtop centrifuge until it became transparent. A black precipitate was obtained on the bottom of centrifuge tube, and the supernatant was removed. Ultrapure water (50 µL) was added to redissolve the burgundy colloidal gold. The above solution was centrifuged for 8 min at 6000 rpm under 4 °C. The upper phase was removed and 50 µL of ultrapure water was added. After that, 1 μL of MCH solution was added for blocking for 20 min. After blocking, the solution was centrifuged and washed three times. Finally, 50 µL of resuspension buffer was added. The resuspension buffer was prepared by weighing 40 mg of Na_3_PO_4_-12H_2_O, 200 mg of bovine serum albumin, 600 mg of sucrose, followed by the addition of 10 µL of Tween-20, and finally the addition of ultrapure water to achieve a total volume of 10 mL.

### 4.5. Preparation of Streptavidin-Biotinylated Cap-Apt and Streptavidin-Biotinylated Con-DNA Conjugates

The procedure involves taking 12 μL of an 80 μmol biotin-modified aptamer capture probe and adding it to 18 μL of a streptavidin (1 mg/mL) solution. The total volume is 30 μL, and the mixture is shaken at 25 °C for 2 h. Similarly, 12 μL of an 80 μmol biotin-Con-DNA is added to 18 μL of a streptavidin (1 mg/mL) solution. The total volume is also 30 μL, and this mixture is shaken at 25 °C for 2 h [34].

### 4.6. Preparation of Lateral Flow Aptasensors

The lateral flow aptasensor comprises four components: an absorbent pad, a nitrocellulose membrane, a conjugate pad, and a sample pad. The absorbent pad was cut into 18 mm × 300 mm, the conjugate pad was cut into 12 mm × 300 mm, and the sample pad was cut into 14 mm × 300 mm. The sample pad and conjugate pad were soaked in a buffer containing 2.5% Tween-20, 0.25% Triton X-100, and 0.05 M Tris-HCl. After treatment, they were dried at 37 °C for 12 h and stored at 4 °C. The control and test zones of the lateral flow aptasensors were prepared by spraying specified volumes of streptavidin-biotinylated Con-DNA (Con-DNA refers to the Control Probe DNA immobilized at the control zone) and streptavidin-biotinylated Cap-aptamer (test zone) solutions onto 25 mm × 300 mm nitrocellulose membranes, respectively. This means that assuming the volumes of both the streptavidin-biotinylated Con-DNA (control zone) and streptavidin-biotinylated Cap-aptamer (test zone) solutions are 30 μL, then there is 1 μL of the streptavidin-biotinylated Con-DNA (control zone) and streptavidin-biotinylated Cap-aptamer (test zone) solution, respectively, on each centimeter of the nitrocellulose membranes. The test and control zones were separated by approximately 4 mm, with the control zone positioned near the absorbent pad. Following this, the nitrocellulose membrane was dried at 37 °C for a duration of 2 h. Subsequently, it was affixed to a PVC bottom plate (60 mm × 300 mm). The conjugate pad, sample pad, and absorbent pad were then fixed sequentially, with each section overlapping by approximately 2 mm to ensure the sample solution could flow through all sections. Finally, the strips were cut into 3.0 mm widths using a CNC strip cutter CTS 300. There is 0.3 μL of the streptavidin-biotinylated Con-DNA (control zone) solution and streptavidin-biotinylated Cap-aptamer (test zone) solution on each test strip.

### 4.7. Assay Procedure

The AFP antigen was diluted with running buffer (10 mM HEPES, 100 mM NaCl, 5 mM glucose, 2 mM MgCl_2_, 0.1% BSA) to varying concentrations, and 70 μL of the solution was applied to the sample pad of the lateral flow aptasensor. Capillary action allows for the sample solution to flow along the test strip, making the detection and control bands visible to the naked eye within 10 min. For quantitative analysis, the intensities of the detection and control bands were assessed using a portable colloidal gold analyzer.

## 5. Conclusions

In this study, a lateral flow aptasensor was developed for rapid detection of the liver cancer tumor marker alpha-fetoprotein (AFP) with a naked-eye detection limit of 10 ng/mL. It is the first report of an antibody-free lateral flow assay using aptamers as recognition probes for the detection of AFP. The biosensor is capable of differentiating the serum samples of healthy individuals from those of patients with liver cancer, which is of great significance for biomedical diagnostics and clinical applications. The subsequent phase of this study will concentrate on enhancing the sensitivity of the assay and selecting a greater number of serum samples from patients and healthy donors for testing, with the objective of increasing the potential for clinical diagnosis.

## Figures and Tables

**Table 1:** Sequences of AFP aptamers.

Note	Sequence (5′–3′)
AP-Taiwan	GGCAGGAAGACAAACAAGCTTGGCGGCGGGAAGG TGTTTAAATTCCCGGGTCTGCGTGGTCTGTGGTGCTGT
AP-273	GTGACGCTCCTAACGCTGAC**TCAGGTGCAGTTCTCGA** **CTCGGTCTTGATGTGGGT**CCTGTCCGTCCGAACCAATC
AP-3	**TCAGGTGCAGTTCTCGACTCGGTCTTGATGTGGGT**

The bold parts in the table represent the same sequences.

## Data Availability

The data that support the findings of this study are available from the corresponding authors upon reasonable request.

## Supplementary Materials

The following supporting information can be downloaded at: https://www.mdpi.com/article/10.3390/molecules30030484/s1, Figure S1: Characterization of AuNP; Figure S2: Gel electrophoresis image of AuNP before and after conjugation with Det-Apt; Figure S3: The optimization of experimental conditions. Figure S4: Pictures and corresponding responses of the tested strips in the specificity test. Figure S5: Pictures and corresponding responses of the tested strips in the detection of real samples. Figure S6: (A) Actual images of the conjugate. The conjugate is dispersed in water (left), and the conjugate is dispersed in the resuspension (right). (B) Particle size diagram of the conjugate dispersed in the resuspension. (C) Electron microscopy image of the conjugate dispersed in the resuspension. Figure S7: Detection of Alpha-fetoprotein (AFP) in Different Loading Buffer Systems. Table S1: Commercial lateral flow AFP test strips. Table S2: Summary of AFP detection by lateral flow biosensors. References [35,36,37,38,39,40,41] are referenced in the Supplementary Materials.
