# Supplementary material for: Rapid Detection of Alpha-Fetoprotein (AFP) with Lateral Flow Aptasensor
Source: Molecules. 2025 Jan 22;30(3):484. doi: 10.3390/molecules30030484 (PMC11820797; doi:10.3390/molecules30030484)
Supplement: Supplementary file 1 [file molecules-30-00484-s001.zip › molecules-3399140-supplementary.pdf]

Supplementary Materials

Rapid detection of alpha-fetoprotein (AFP) with lateral flow Aptasensor

Meijing Ma[1], Min Zhang[1], Jiahui Wang[1], Yurui Zhou[1], Xueji Zhang[2]*, Guodong Liu[1]*,

[1] School of Chemistry and Chemical Engineering, Linyi University, Linyi 276005, China; 220703001552@lyu.edu.cn (M.M.); 220703001572@lyu.edu.cn (M.Z.); wangjiahui1@lyu.edu.cn (J.W.); zhouyurui@lyu.edu.cn (Y.Z.)
2 Marshall Laboratory of Biomedical Engineering, Research Center for Biosensor and Nanotheranostic, School of Biomedical Engineering, Shenzhen University, Shenzhen 518060, China
* Correspondence: zhangxueji@szu.edu.cn (X.Z.); liuguodong2@lyu.edu.cn (G.L.)

1. Characterization of AuNP and AuNP-Apt conjugate

Figure S1(A) depicts a representative transmission electron microscopy (TEM) image of gold nanoparticles (AuNPs). **Figure S1(B)** illustrates the particle size distribution of the AuNPs. **Figure S1(C)** displays the ultraviolet (UV) spectra of the gold nanoparticles before and after conjugation with the AFP aptamer. The alteration in the peak shape around 263 nm indicates the successful conjugation of the AFP aptamer[24].

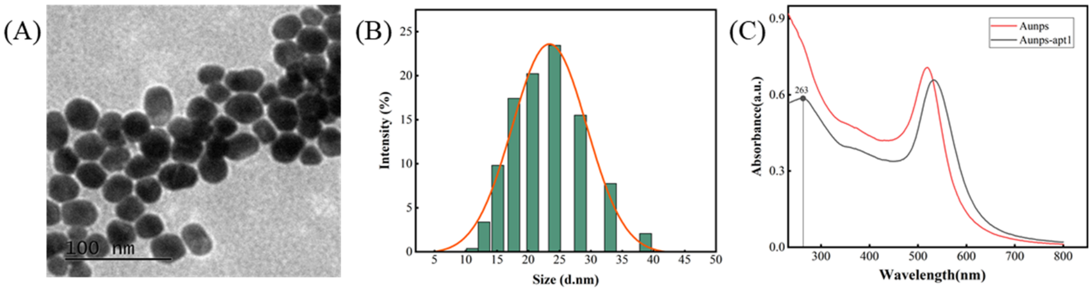

Figure S1. Characterization of AuNP: (**A**) TEM image of AuNPs with a diameter of 25 nm. (**B**) Size distribution diagram of AuNPs. (**C**) UV absorption spectra before and after conjugation.

2. Gel electrophoresis assays of AuNP-Det-Apt conjugates

Firstly, 1.5% agarose gel was prepared. Subsequently, 5 μl of AuNP solution and AuNP-Det-Apt conjugate were added to the spiking wells, respectively. Following this, an electrophoretic test was carried out in TBE buffer solution for 30 minutes, resulting in the experimental results, as illustrated in **Figure S2**. The image demonstrates that AuNP does not generate bands in the experimental setting. Additionally, the colour of the AuNP changed from red to black in the spiked wells. Conversely, the AuNP-Det-Apt conjugate produces red bands and retains its colour after being introduced into the sample wells. This evidence substantiates the successful conjugation of the AFP aptamer.

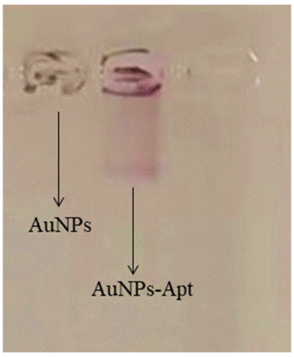

Figure S2. Gel electrophoresis image of AuNP before and after conjugation with Det-Apt.

3. Optimization of analytical parameters

The preparation and experimental parameters of aptamer-based lateral flow biosensors have a significant impact on its analytical performance. To achieve optimal reproducibility and sensitivity, we optimized several parameters: (a) the AuNP concentration, (b) the type of nitrocellulose membrane, (c) the concentration of aptamers during conjugation, (d) the volume of AuNP-Det-Apt conjugates on the conjugate pad, (e) the volume of the running buffer, and (f) the volume of streptavidin-biotinylated Cap-Apt (Capture probe aptamer) in the test zone. The analytical performance of lateral flow aptasensor was assessed using the signal-to-noise ratio (S/N), with one condition optimized at a time while keeping the others constant. A comprehensive analysis of the collected data, along with the relevant graphs, is provided in **Figure S3**. The experimental conditions used to achieve the best results were as follows: (a) the optimal concentration of AuNP is 16 OD., (b) VIVID nitrocellulose mem-branes were found to be the most effective, (c) the optimal concentration of the aptamer during conjugation is 8 μmol, (d) the optimal volume of conjugate required at each strip conjugate pad is 1.3 μL, (e) a volume of 50 μL of running buffer has been found to be optimal, (f) the volume of streptavidin biotinylated cap-DNA present within the designated test area is 1.5 μL.

In order to achieve the optimal colour development effect, the colloidal gold solution is typically concentrated prior to conjugation. Therefore, the colloidal gold solution was concentrated to different concentrations and compared. As illustrated in **Figure S3(a)**, The best results can be obtained when the concentration of AuNP is 16 OD. This is due to the fact that the colour development is poor when the concentration is low, whereas too high a concentration results in a slight false-positive background signal, leading to a decrease in the signal-to-noise ratio instead.

The pore size of different nitrocellulose membranes varies, which consequently affects the flow rate of the up-sampling buffer and the conjugation efficiency of AFP-apt. Consequently, we selected VIVID, CN140 and JN120m membranes for optimisation.

The full name of the VIVID membrane is Pall vivid 90. Its flow rate is usually 80-100 seconds per 25 mm. It has a stable protein-binding capacity, which can meet general testing requirements and performs well in food safety testing.

The full name of the CN 140 membrane is Sartorius CN 140. It is manufactured by Sartorius. With a pore size of 10 μm and a capillary flow rate of 95-155 seconds per 40 mm, it is a membrane with high sensitivity and is often used in various lateral-flow diagnostic analyses. It has

a strong protein-binding ability and can firmly adsorb proteins. In the immune reaction, it can better immobilize antibodies or antigens, ensuring the accuracy and sensitivity of the test.

The JN120m is made by Shanghai Jiening Biotechnology Co., Ltd. The pore size and chromatographic speed performance are between the above-mentioned two kinds of membranes. JN120 m usually undergoes strict quality control during the production process as well to reduce background interference. It can be applied in a variety of biological detection fields, such as immunochromatography, biosensors and so on.

Figure S3(b) demonstrates that the VIVID membrane was the optimal choice for detection.

Subsequently, the concentration of the aptamer probe and the volume of the conjugate dropped onto the conjugate pad were optimized during the conjugation process. **Figure S3(c)** shows that the signal-to-noise ratio gradually increases with the increase in the probe concentration, reaching a peak when the concentration is 8 μmol. **Figure S3(d)** demonstrates that an increase in the volume of conjugate at the conjugate pad results in a decline in the signal-to-noise ratio. This phenomenon can be attributed to the fact that when colloidal gold adsorbs a greater number of detection probes, the conjugation efficiency is enhanced, leading to an improved signal-to-noise ratio. However, an excess of conjugate volume at the conjugate pad can result in non-specific adsorption. Consequently, an increase in the volume of conjugate at the conjugate pad results in a reduction in the signal-to-noise ratio.

Figure S3(e) illustrates that the optimal volume of the up-sampling buffer is 50 μL. This is due to the fact that when the volume of the up-sampling buffer is excessive, the T/C line region develops colour and subsequently exhibits a slight diffusion phenomenon with the increase of solution, resulting in a slightly lighter colour signal. The delineation concentration of the capture probe was also optimised, as this affects several key parameters, including non-specific adsorption, hybridisation efficiency between the AFP protein and the detection aptamer probe, and sensitivity of the assay. As can be seen from **Figure S3(f)**, the optimal signal-to-noise ratio was obtained at a delineation concentration of 1.5 μL/cm.

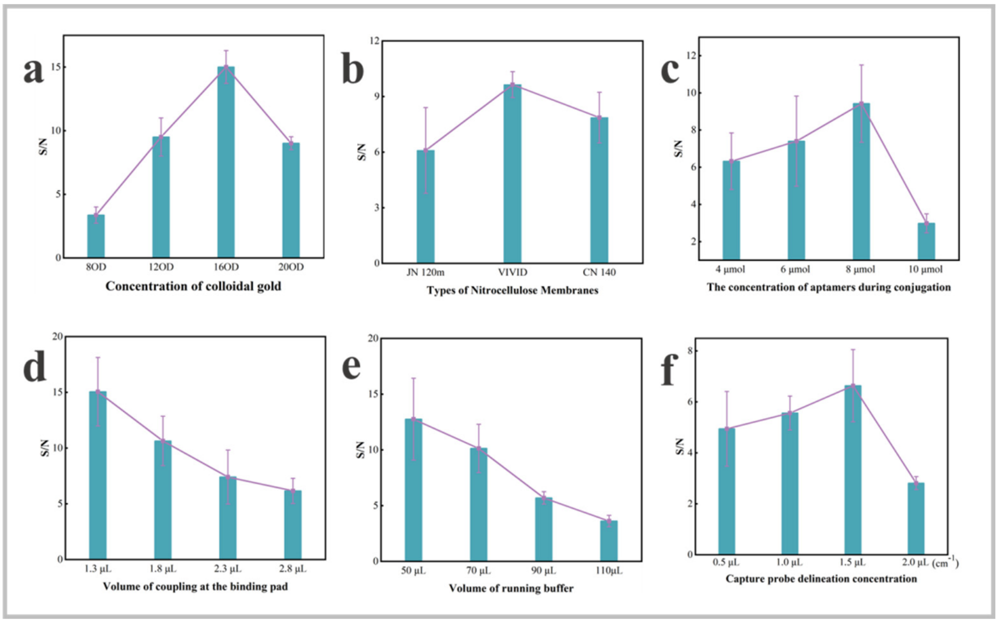

Figure S3. The optimization of experimental conditions. **(a)** the AuNP concentration, **(b)** the type of nitrocellulose membrane, **(c)** the concentration of aptamers during conjugation, **(d)** the volume of

AuNP-Det-Apt conjugates on the conjugate pad, **(e)** the volume of the running buffer, and **(f)** the volume of streptavidin-biotinylated Cap-Apt (Capture probe aptamer) in the test zone.

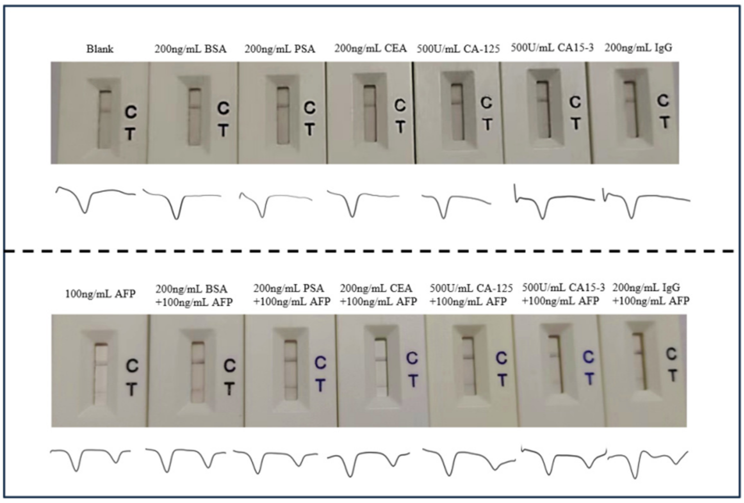

Figure S4. Pictures and corresponding responses of the tested strips in the specificity test.

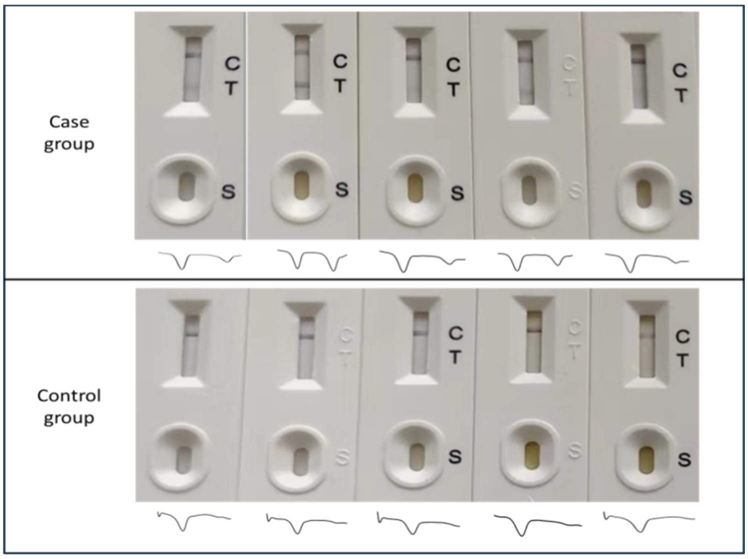

Figure S5. Pictures and corresponding responses of the tested strips in the detection of real samples.

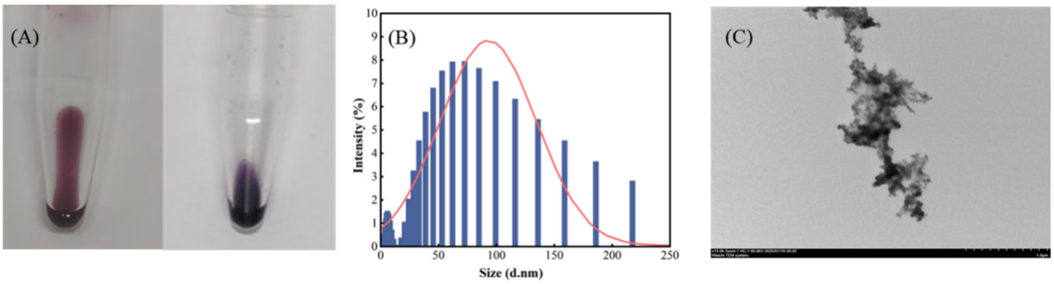

Figure S6(A) Actual images of the conjugate. The conjugate is dispersed in water (left), and the conjugate is dispersed in the resuspension (right). Figure S6(B) Particle size diagram of the conjugate dispersed in the resuspension. Figure S6(C) Electron microscopy image of the conjugate dispersed in the resuspension.

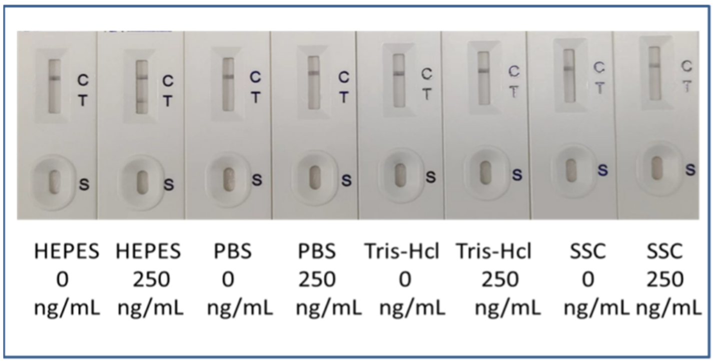

Figure S7. Detection of Alpha - fetoprotein (AFP) in Different Loading Buffer Systems

Table S1. Commercial lateral flow AFP test strips

Company	Name	Method	Detection Limit
Tianjin Zhongxinkeju Biopharmaceutical Co., Ltd.	Alpha-Fetoprotein (AFP) Detection Kit	Colloidal Gold Method	20 ng/ml
Guangzhou Jian lun Biotechnology Co., Ltd.	Alpha-Fetoprotein (AFP) Assay Reagent	Colloidal Gold Method	20 ng/ml
Nantong Yishi Biotechnology Co., Ltd.	Alpha-Fetoprotein Detection Kit	Colloodal Gold Method	-
Xiamen Bo sheng Biotechnology Co., Ltd.	Alpha-Fetoprotein (AFP) Assay Kit	Colloidal Gold Method	-

NOVA test	AFP Rapid Test Kits	Colloidal Gold Method	-
Accuquik	AFP Test Kits	Colloidal Gold Method	-
Vitrosens Biotechnology	AFP Rapid Test Kits	Colloidal Gold Method	-
Fortress Diagnostics	AFP Rapid Diagnotical Test	Colloidal Gold Method	-
Creative Diagnostics	Alpha-Fetoprotein Rapid Test	Colloidal Gold Method	0.18 ng/ml

Table S2. Summary of AFP detection by lateral flow biosensors

Substances to be detected	biomarker molecules	material	signal type	LOD
AFP	Antibody	Silica/CdTe quantum dots	fluorescence	2 ng/ml [12]
AFP	Antibody	Carboxyl modified polystyrene microspheres	fluorescence	0.1IU/ml [35]
AFP	Antibody	Au-HRP	chemical luminescence	0.21 ng/mL [36]
AFP	Antibody	AuNP@SiO_2	visible light	0.3 ng/mL [37]
AFP	Antibody	raspberry –like Fe_3O_4@Au MNPs	raman signal	1pg /mL [38]
AFP	Antibody	RENPs (NaYF4:Nd,Yb)	near infrared light	2 ng/mL [15]
AFP	Antibody	Yb, Er/Ceco-doped core-shell nanoparticles	near infrared light	1.00 ng/mL [39]
AFP	Antibody	NIR-II AIE pigmentBBTD@PS	near infrared light	0.24 ng/mL[40]
AFP	Antibody	Hollow Gold Nanocages	heat	0.25 ng/mL [41]
AFP	Aptamer	AuNP (This paper)	visible light	10 ng/mL

References

12. Bai, Y.; Tian, C.; Wei, X.; Wang, Y.; Wang, D.; Shi, X. A sensitive lateral flow test strip based on silica nanoparticle/CdTe quantum dot composite reporter probes. *RSC Adv.* **2012**, *2*, 1778–1781.
15. Liu, Q.; Cheng, S.; Chen, R.; Ke, J.; Liu, Y.; Li, Y.; Feng, W.; Li, F. Near-infrared lanthanide-doped nanoparticles for a low interference lateral flow immunoassay test. *ACS Appl. Mater. Interfaces* **2020**, *12*, 4358–4365.
24. Schmid, F.X. Biological Macromolecules: UV-visible Spectrophotometry. Encyclopedia of Life Sciences, 2001. DOI: 10.1038/ npg. els. 0003142.
35. Liang, R.-L.; Xu, X.-P.; Liu, T.-C.; Zhou, J.-W.; Wang, X.-G.; Ren, Z.-Q.; Hao, F.; Wu, Y.-S. Rapid and sensitive lateral flow immunoassay method for determining alpha fetoprotein in serum using europium (III) chelate microparticles-based lateral flow test strips. *Anal. Chim. Acta* **2015**, *891*, 277–283.
36. Chen, Y.; Sun, J.; Xianyu, Y.; Yin, B.; Niu, Y.; Wang, S.; Cao, F.; Zhang, X.; Wang, Y.; Jiang, X. A dual-readout chemiluminescent-gold lateral flow test for multiplex and ultrasensitive detection of disease biomarkers in real samples. *Nanoscale* **2016**, *8*, 15205–15212.
37. Lu, X.; Mei, T.; Guo, Q.; Zhou, W.; Li, X.; Chen, J.; Zhou, X.; Sun, N.; Fang, Z. Improved performance of lateral flow immunoassays for alpha-fetoprotein and vanillin by using silica shell-stabilized gold nanoparticles. *Microchimica Acta* **2019**, *186*, 1–7.
38. Shen, W.; Wang, C.; Yang, X.; Wang, C.; Zhou, Z.; Liu, X.; Xiao, R.; Gu, B.; Wang, S. Synthesis of raspberry-like nanogapped Fe 3 O 4@ Au nanocomposites for SERS-based lateral flow detection of multiple tumor biomarkers. *J. Mater. Chem. C* **2020**, 8, 12854–12864.
39. Li, Y.; Ke, J.; Liu, Q.; Yuan, W.; Su, Q.; Kong, M.; Wu, N.; Feng, W.; Huang, C.; Li, F. NIR-II emitting rare-earth nanoparticles for a lateral flow immunoassay in hemolysis. *Sens. Actuators B: Chem.* **2021**, *345*, 130380.
40. Chen, R.; Zhou, X.; Wu, Y.; Liu, Q.; Liu, Q.; Huang, J.; Li, F. NIR-II emissive lateral flow immunoassay for accurate determination of tumor marker in hemolysis. *Sens. Actuators B: Chem.* **2021**, *328*, 129050.
41. Hu, X.; Wan, J.; Peng, X.; Zhao, H.; Shi, D.; Mai, L.; Yang, H.; Zhao, Y.; Yang, X. Calorimetric lateral flow immunoassay detection platform based on the photothermal effect of gold nanocages with high sensitivity, specificity, and accuracy. *Int. J. Nanomedicine* **2019**, 7695–7705.
